# Effect of essential phospholipids on hepatic steatosis in metabolic dysfunction-associated steatotic liver disease associated with type 2 diabetes mellitus and/or hyperlipidemia and/or obesity: study protocol of a randomized, double-blind, phase IV clinical trial

**DOI:** 10.1186/s13063-024-08208-4

**Published:** 2024-06-11

**Authors:** Norbert Stefan, Marek Hartleb, Branko Popovic, Rafael Varona

**Affiliations:** 1 Department of Internal Medicine IV, University Hospital Tübingen, Tübingen, Germany; 2 Department of Gastroenterology and Hepatology, Faculty of Medicine, Medical University of Silesia, Katowice, Poland; 3 Sanofi, Frankfurt am Main, Germany; 4 Sanofi, Neuilly-sur-Seine, France

**Keywords:** MASLD, EPL, Hyperlipidemia, T2DM, Standard of care, Quality of life

## Abstract

**Background:**

Metabolic dysfunction-associated steatotic liver disease (MASLD) is a predominant chronic liver condition globally and is strongly associated with obesity, diabetes mellitus, and dyslipidemia. Essential phospholipids (EPL) are recommended as supportive treatment for managing liver conditions, including MASLD or metabolic dysfunction-associated steatohepatitis, cirrhosis, and viral hepatitis. While efficacy of EPL as an adjunctive therapy in MASLD treatment has been established earlier, certain aspects of its usage such as the impact of standard-of-care parameters, effect of EPL on quality of life (QoL) and change in symptoms evaluation in patients with MASLD remain unexplored. The proposed trial aims to assess the efficacy and safety of EPL and the subsequent QoL of patients with MASLD associated with type 2 diabetes mellitus (T2DM) and/or hyperlipidemia and/or obesity.

**Methods:**

This is a multicenter, multinational, double-blind, randomized, two-arm, placebo-controlled, parallel-group, phase IV clinical trial. The trial is being conducted in approximately 190 patients who are randomized on a 1:1 basis either to the EPL arm (Essentiale® 1800 mg/day orally + standard of care) or placebo arm (placebo + standard of care). The primary outcome is to assess the efficacy of EPL on hepatic steatosis, as measured by transient elastography, from baseline to 6 months. The secondary outcomes include change in QoL parameters, as measured by the Chronic Liver Disease Questionnaire–metabolic dysfunction-associated steatotic liver disease/ metabolic dysfunction-associated steatohepatitis and change in symptom evaluation (using the Global Overall Symptom scale) from baseline to 6 months for symptoms, including asthenia, feeling depressed, abdominal pain/discomfort, or fatigue.

**Discussion:**

The current protocol design will allow to comprehensively explore the efficacy of EPL added to the standard of care on hepatic steatosis and QoL and its safety in patients with MASLD associated with T2DM and/or hyperlipidemia and/or obesity by assessing various outcome measures.

**Trial registration:**

European Union Clinical Trials Register, EudraCT, 2021–006069-39. Registered on March 13, 2022.

**Supplementary Information:**

The online version contains supplementary material available at 10.1186/s13063-024-08208-4.

## Introduction

Metabolic dysfunction-associated steatotic liver disease (MASLD) formerly known as non-alcoholic fatty liver disease (NAFLD) is the leading chronic liver condition globally, affecting around 30–32% of the population [1, 2]. It encompasses various liver abnormalities, mainly characterized by excess fat accumulation due to obesity, diabetes, and dyslipidemia [3–6]. MASLD not only increases mortality [7, 8] but also impacts patients’ quality of life negatively (QoL) [9].

A consensus statement on a revised nomenclature for a fatty liver disease was recently published by multiple societies using the Delphi method. This statement introduced the term MASLD and effectively replacing NAFLD [10].

Early-stage steatosis in MASLD poses risks for disease progression and other metabolic disorders like obesity, hypertension, type 2 diabetes mellitus (T2DM), and cardiovascular diseases, especially when associated with metabolic dysfunction-associated steatohepatitis (MASH) and liver fibrosis [11–14].

Traditionally, diagnosing hepatic steatosis in MASLD required invasive liver biopsy. However, noninvasive imaging techniques like transabdominal ultrasonography and FibroScan, which assess liver stiffness, provide alternatives with advantages in affordability, noninvasiveness, and widespread accessibility [15–17]. FibroScan utilizes ultrasound-based elastography to detect liver fibrosis [18]. Further, a controlled attenuation parameter (CAP) combines FibroScan with the assessment of liver stiffness, enabling simultaneous evaluation of both liver fibrosis and severity of MASLD [19].

In MASLD, particularly MASH, levels of polyunsaturated phosphatidylcholine (PC) are lower compared to healthy individuals, affecting liver cell function [20, 21]. Essentiale®, a medicinal product that contains de-oiled, enriched phospholipids extracted from soybean, with 72% of 3-sn-phosphatidyl-choline (also known as essential phospholipids [EPL]), helps normalize lipid and protein metabolism, improve liver detoxification, restore cellular structure, and delay of conjunctive tissue production [22–24]. In Europe, EPL are indicated for improvement of subjective symptoms in patients with liver damage due to chronic liver disease, liver cirrhosis, fatty liver, and intoxication by hepatotoxic substances [22].

The safety of EPL (study drug) has been well established since several decades. Post-marketing observations suggest a safety profile comparable with other products in the therapeutic class [23]. The identified risks include gastrointestinal disorders (in the form of stomach disorders and/or diarrhea), skin and subcutaneous tissue disorders such as allergic reactions (e.g., skin rash or exanthema, urticaria) or pruritus. However, their frequency remains mostly unknown due to limited data. Despite this, the overall benefit-risk assessment of the study drug remains positive under current approved usage conditions [22].

Several studies have investigated the effectiveness of EPL in liver pathologies such as steatosis (caused by MASLD or alcohol-related liver diseases), cirrhosis, fulminant hepatitis, toxic liver injury, and acute and chronic viral hepatitis [24, 25], as well as cholestasis and cholelithiasis [26]. Several randomized controlled clinical trials have focused on the effects of EPL in the treatment of MASLD [27]. A double-blind, placebo-controlled study demonstrated that patients with diabetes and fatty liver receiving EPL for over 6 months experienced a significant reduction in liver size; the mean serum gamma-glutamyl transferase (GGT) activity was significantly lower after 1, 3, and 6 months of follow-up than baseline levels [27]*.* Recently, the MANPOWER product registry investigated the effects of EPL as an adjunctive treatment alongside standard of care in patients with newly diagnosed MASLD who also had at least one of the following four comorbidities: overweight/obesity, hypertension, T2DM, and/or hypercholesterolemia. A majority (99.8%) of these patients received EPL. After 24 weeks of treatment, significant improvements were observed in liver echogenicity (68.3%) and liver structure (42.7%) compared with baseline [28].

While efficacy of EPL as an adjunctive therapy in MASLD treatment has been established earlier, certain aspects of its usage remain unexplored [29–31]. In a prospective, randomized, open-label study, although a significant improvement in clinical parameters and transaminase levels was observed in patients with MASLD receiving EPL, the impact of standard-of-care parameters such as diet and exercise could not be determined [29]. In an observational, multicenter study with patients having MASLD and at least one of the four comorbidities (overweight/obesity, hypertension, T2DM, and hypercholesterolemia), treatment with EPL resulted in high levels of treatment adherence and satisfaction. However, liver biopsy and elastography were rarely performed to determine different stages of steatosis in MASLD and differentiation between MASLD stages was achieved using available data from ultrasonography and liver enzyme activity. Further, the study could not assess patients’ adherence to combined EPL therapy and comorbidity-related medications because of a decreased rate of concomitant medications and patients’ noncompliance to dietary restrictions [30].

Similarly, the effect of EPL on QoL, change in symptoms (such as sleeping disorder, fatigue, and abdominal pain), and evaluation in patients with MASLD has not been thoroughly investigated yet.

The aim of this clinical trial is to investigate the efficacy and safety of EPL in patients with MASLD associated with T2DM and/or hyperlipidemia and/or obesity. The effect of EPL on liver steatosis, as measured by a noninvasive technique—CAP as well as changes in liver stiffness—would be evaluated. QoL parameters, as measured by the Chronic Liver Disease Questionnaire–metabolic dysfunction-associated steatotic liver disease/ metabolic dysfunction-associated steatohepatitis (CLDQ-MASLD/MASH), a validated questionnaire specific for MASLD, will also be assessed.

## Methods

### Clinical trial design

This phase IV clinical trial is a multicenter, multinational study following a double-blind, randomized, two-arm, placebo-controlled, parallel-group design. The study is being conducted in patients with MASLD associated with T2DM and/or hyperlipidemia and/or obesity, willing to follow lifestyle modification related to diet and physical activity/exercise throughout the trial period.

Patients are being randomly assigned in a 1:1 ratio to one of the two treatment arms: EPL arm (Essentiale® 1800 mg/day orally + standard of care) or placebo arm (placebo + standard of care).

During this study, the investigator records the standard-of-care recommendation (e.g., physical activity/exercise recommendation, diet restriction, alcohol restriction, and coffee recommendation) for each patient in an electronic case report form (eCRF). To ensure that patients consistently adopt and maintain lifestyle modifications throughout the trial, the study sites are encouraged to implement the standard-of-care guidelines that are tailored to their specific site and disease requirements, or alternatively, to follow the 2016 European Association for the Study of the Liver (EASL) guidelines [32].

**Fig. 1:**
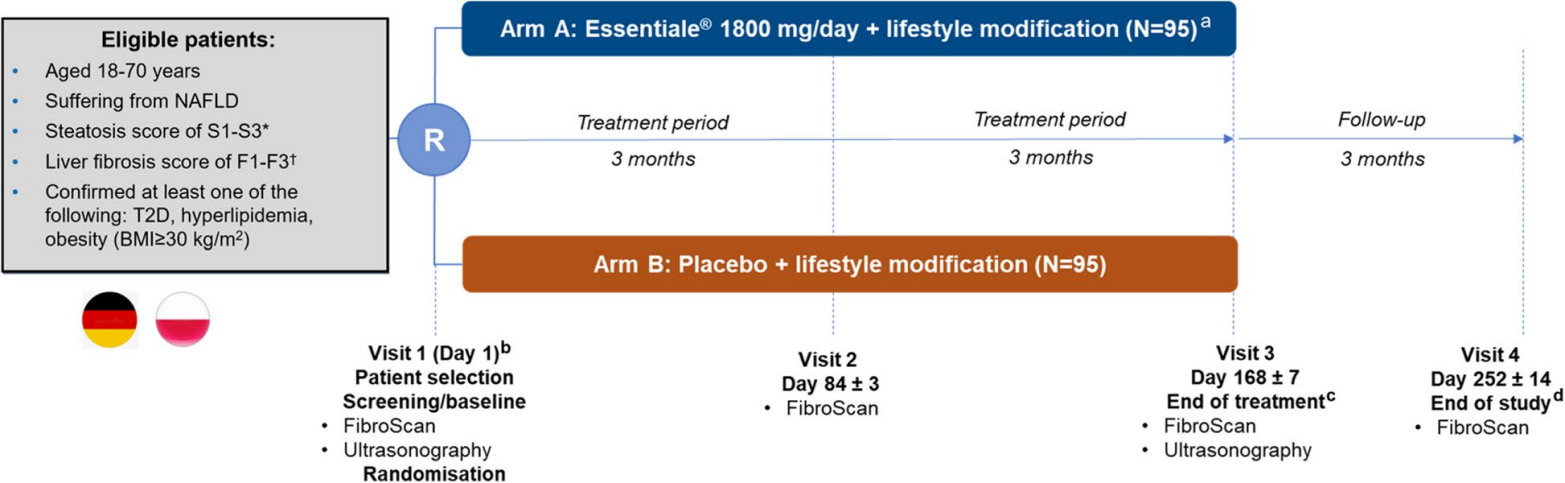
Study design

The trial consists of a patient enrollment visit—Visit 1 (encompasses screening and baseline) of up to 2 days, control visits after 3 and 6 months of the treatment—Visits 2 and 3, and an end-of-trial visit after a 3-month, and post-treatment follow-up period—Visit 4 (Fig. 1).

During Visit 1, day 1 of the study, all eligible patients undergo assessments, including transient elastography (FibroScan) and ultrasonography, as part of the patient enrollment visit. They are being randomly assigned to the EPL arm or the placebo arm and have initiated their study drug treatment. These procedures aim to evaluate liver health and establish a baseline for comparison during the study.

Patients undergo transient elastography (FibroScan) at Visit 2, day 84 ± 3 (control visit), at Visit 3, day 168 ± 7 (end-of-treatment visit), and at Visit 4, day 252 ± 14 (end-of-study visit). Assessment of patients’ QoL, as measured by the CLDQ-MASLD/MASH, and severity of symptoms (using the Global Overall Symptom [GOS] scale) will be assessed at the time points specified in the schedule of events.

Patients undergo ultrasonography at Visit 3, day 168 ± 7 (end-of-treatment visit); blood samples will be collected for measuring liver enzyme levels (ALT, AST, and GGT) and lipid levels (low-density lipoprotein [LDL], high-density lipoprotein [HDL], triglycerides, and total cholesterol; fasting samples), glycemic index (HOMA-IR; using fasting plasma glucose and insulin values), and glycated hemoglobin (HbA1c) at time points specified in the schedule of events.

Patient satisfaction with effectiveness, as measured by a 4-point Likert scale, and patient intention of recommending the treatment are assessed as exploratory endpoints. Adverse events will be assessed. Physical examination and clinical safety laboratory tests are conducted, and vital signs are measured to evaluate the safety of the study drug.

**Fig. 2:**
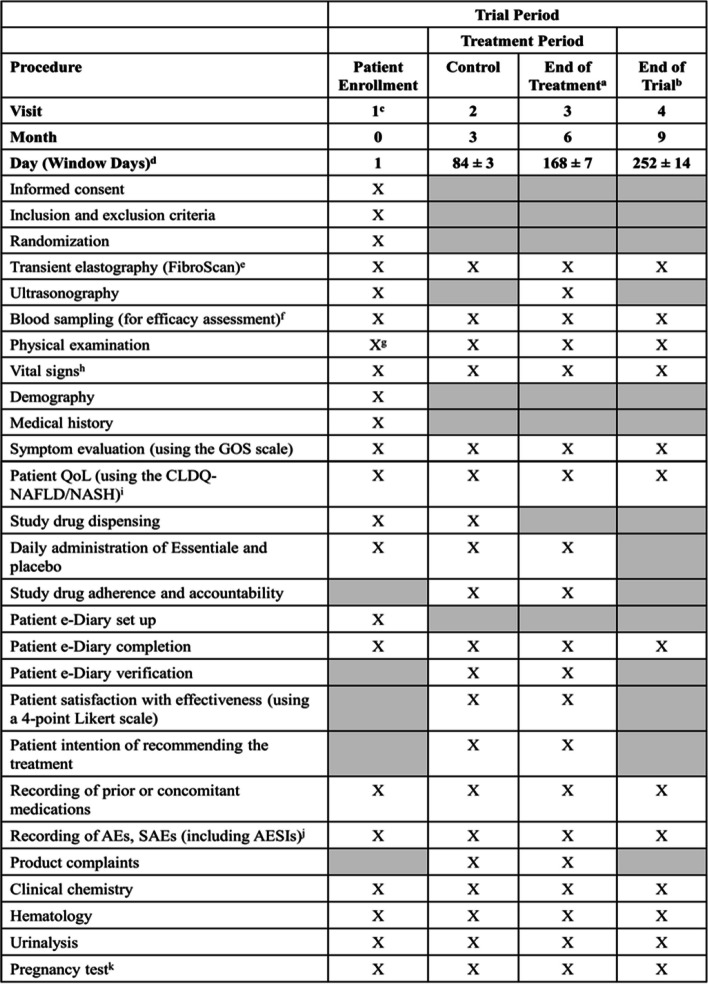
Schedule of events

A urine pregnancy test (only for women of childbearing potential) will be performed at the time points specified in the schedule of events (Fig. 2). Any female patient who tested positive for pregnancy while participating in the trial will be discontinued from study drug treatment and this would be reported as AESI.

The maximum duration of the trial for a patient is approximately 10 months (1 month = 28 days), including a time interval of up to 28 days for the patient enrollment visit (if performed on two different days).

The protocol was reported in accordance with the Standard Protocol Items: Recommendations for Interventional Trials guidelines [33]. Protocol version at time of writing is LPS16141 (Amendment 2, Version 3.0). For a completed SPIRIT checklist, see Additional file 1.

### Trial registration

EudraCT, 2021–006069-39. Registered on 13 March 2022, European Union Clinical Trials Register [34].

### Ethical approval

This study was approved by the Ethics Committee at the Medical Faculty of the Eberhard Karls University and at the University Hospital Tübingen, Germany (Project Number: 251/2022AMG1). The trial is being performed in accordance with the ethical principles that have their origin in the Declaration of Helsinki, the International Council for Harmonisation of Technical Requirements for Pharmaceuticals for Human Use (ICH) Guideline for Good Clinical Practice (GCP), and all applicable regulations. Written consent is obtained from each participant prior to the collection of data.

### Participants

Approximately 190 patients are enrolled at several sites in Poland and Germany. Patients are expected to sign written informed consent, complete the electronic diary (eDiary), and agree to comply with the protocol requirements.

#### Inclusion criteria

- Male or female adult (18–70 years) patients, with a confirmed diagnosis of MASLD and a steatosis score of S1–S3 (defined as CAP score > 248 dB/m, as measured by transient elastography) and a liver fibrosis score of F1–F3 (defined as liver stiffness measurement [LSM] of 5–13 kPa, as measured by transient elastography).
- The patients should have a confirmed diagnosis of at least one of the following associated illnesses.
  - ➢ T2DM treated with diabetes medications (e.g., metformin and insulin), with stable doses for 3 months before the patient enrollment visit and willingness to continue medications during the trial.
  - ➢ Hyperlipidemia treated with medications such as statins, with stable doses for 3 months before the patient enrollment visit and willingness to continue medications during the trial.
  - ➢ Obesity (defined as body mass index [BMI] ≥ 30 kg/m^2^).

#### Exclusion criteria

- Patients with other causes of liver disease or abnormal laboratory results (AST ≥ 4 × upper limit of normal [ULN], ALT ≥ 4 × ULN, bilirubin ≥ 2 × ULN) or cirrhosis within 3 months before the patient enrollment visit.
- Patients with current viral hepatitis or diagnosed with type 1 diabetes mellitus (T1DM) or with an HbA1c > 10.0% within 3 months before the patient enrollment visit.
- Patients with severe heart disease (e.g., heart failure) according to New York Heart Association (NYHA) Functional Classification (Class II–IV; The Criteria Committee of the New York Heart Association 1994) or severe renal impairment, as defined by estimated glomerular filtration rate of < 15 mL/min/1.73 m^2^.
- Patients having current or known history of drug abuse (within 6 months before the patient enrollment visit) or alcohol consumption (> 20 g per day in women or > 30 g per day in men).
- Patients hypersensitive to EPL or its components or any of its excipients that may contraindicate participation in the trial.
- Patients enrolled in another clinical trial or have taken other investigational drug(s) within 1 month before the patient enrollment visit.
- Patients currently hospitalized for planned surgery.

### Randomization, allocation, and (un)blinding

All patients are centrally assigned to randomized treatment arms at Visit 1, day 1 (patient enrollment visit) to receive EPL 300 mg oral capsules or placebo using an Interactive Response Technology (IRT; i.e., interactive voice response system, interactive web response system) with a 1:1 allocation ratio. The randomization schedule is being generated using SAS Enterprise Software Version 7.1 or higher (SAS Institute Inc., Cary, NC, USA) for the IRT, resulting in linking of sequential patient randomization numbers to treatment codes. The randomization schedule was stratified by country and is carried out using an appropriate block size.

This is a double-blind trial. During the trial period, investigators and patients will be blinded to the allocation of the EPL or placebo arms. The sponsor will also be blinded to treatment allocation throughout the trial period. EPL and placebo capsules will be provided in indistinguishable blister packs. In accordance with the double-blind design, investigators will remain blinded to the treatment and will not have access to the randomization (treatment) codes except under exceptional medical circumstances.

The study drug is EPL or matching placebo and will be supplied as a hard capsule for oral administration. Placebo matching capsules will be identical in appearance to Essentiale 300 mg capsules.

During any medical emergency, the individual patient’s treatment allocation may be unblinded by the investigator through the IRT system.

### Discontinuation of study drug

In the event of permanent discontinuation of the study drug, all required assessments requested at the end-of-treatment visit (day 168 ± 7) and at the end-of-trial visit (day 252 ± 14) will be conducted. Patients who withdraw prematurely from the trial will undergo end-of-study visit assessments at an identified early trial discontinuation visit. All cases of permanent study drug discontinuation and patient’s withdrawal must be recorded by the investigator in the appropriate pages of the eCRF when confirmed.

### Adherence

Study drug adherence is being monitored through study drug kit at each visit after starting medication and patients record their daily dose administered in an eDiary.

All patients are expected to take six capsules (300 mg each) of EPL or placebo per day, and adherence is based on the treatment/accountability log by checking the number of capsules provided versus the number of capsules returned (whether empty or unused). Adherence will be calculated using the following formula:

$${\mathrm{Adherence}\;\mathrm{rate}\;\left(\%\right)}=\frac{\text{Total number of EPL or placebo capsules taken}}{\text{Total number of EPL or placebo capsules expected to be taken}}\times100$$

The total number of capsules taken is calculated by summing up each patient’s capsule consumption from Visit 1, day 1 through the date of last dosing day at Visit 3, day 168 ± 7 (or date of discontinuation).

### Concomitant care

Patients are required to record details of their concomitant medication(s) in the eDiary at least once daily. Any concomitant medication deemed necessary for the welfare of the patient during the trial may be given at the discretion of the investigator.

Although there are no specific medications that are contraindicated with the use of EPL, it is possible that there could be interactions between EPL and medications that inhibit blood coagulation. In such cases, it may be necessary to adjust the dosage of the anticoagulant medication. Use of all concomitant medications is recorded in the patients’ diaries and eCRFs.

### Participant timeline

Figure 2 shows the schedule of enrollment, intervention, and assessments during this study in accordance with the SPIRIT statement [33].

### Endpoints

#### Primary endpoint

The primary endpoint is to assess the efficacy of EPL added to standard of care compared with placebo added to standard of care, by examining the change in steatosis from baseline to 6 months (Visit 3), as measured by transient elastography (CAP score). This difference will be compared between the two arms.

#### Secondary endpoints

Secondary endpoints can be divided into several categories:

- Change in QoL total score from baseline to 6 months, as measured by the CLDQ-MASLD/MASH.
- Change in symptom evaluation (using the GOS scale) from baseline to 6 months for the following 4 major symptoms: asthenia, feeling depressed, abdominal pain/discomfort, or fatigue.

#### Exploratory endpoints

- Changes from baseline to 6 months in the following parameters: liver fibrosis, as measured by transient elastography (LSM); ultrasonography; ALT, AST, and GGT levels; HbA1c; and blood lipid levels (LDL, HDL, triglycerides, and total cholesterol).
- Rate of recovery of ALT, AST, GGT; and blood lipid levels (LDL, HDL, triglycerides, and total cholesterol) for abnormal parameters at baseline.
- Change in symptom evaluation (using the GOS scale) from baseline to 6 months for the following three additional symptoms: sleeping disorder, appetite loss, or irritability.
- Changes from baseline to 3 and 9 months in the following parameters: steatosis, as measured by transient elastography (CAP score); liver fibrosis, as measured by transient elastography (LSM); QoL, as measured by the CLDQ-MASLD/MASH; ALT, AST, and GGT levels; HbA1c; glycemia index (Homeostatic Model Assessment for Insulin Resistance [HOMA-IR]); symptom evaluation (using the GOS scale) for asthenia, feeling depressed, abdominal pain/discomfort, and fatigue; and blood lipid levels (LDL, HDL, triglycerides, and total cholesterol).
- Patient satisfaction with effectiveness at 6 months, as measured by a 4-point Likert scale.

### Estimands and intercurrent events

For efficacy analysis, estimand framework will be used in this trial. The possible ICE (intercurrent events) type may be death due to any cause or discontinuation of study drug due to treatment-emergent adverse event. For the primary endpoint of change in steatosis, as measured by transient elastography (CAP score), from baseline to 6 months, the ICE strategy is hypothetical.

### Data management

The investigator is committed to treating data with strict confidentiality and ensuring the accuracy of eCRFs and source documentation, which may comprise patient-reported outcome questionnaires, medical charts, the patients’ eDiary, and laboratory reports, forming essential components of the case histories.

Clinical data management plays a vital role in maintaining data integrity by addressing errors and inconsistencies. It adheres to relevant trial standards and employs data cleaning procedures to ensure accuracy. The Medical Dictionary for Regulatory Activities (MedDRA) and WHODrug will be utilized for coding adverse events and concomitant medication terms, respectively. Once the database is locked, each site receives their site-specific eCRF data from Medidata Rave, including full discrepancy and audit history. Additionally, a copy of all data from the trial will be created and sent to the sponsor for storage.

### Statistical analyses

#### Primary analysis

For the primary endpoint of change in the CAP score, as measured by transient elastography, from baseline to 6 months between the EPL arm and the placebo arm, the following statistical null and alternative hypothesis will be tested:

- H0: μA = μR.
- H1: μA ≠ μR.

where μX is change in the CAP score from baseline at month 6 for the treatment arms: EPL added to standard of care (X = A) and placebo added to standard of care (X = R). The planned statistical analysis involves utilizing a mixed-effects model with repeated measures (MMRM) to examine the hypothesis. The MMRM will incorporate several fixed categorical effects, including treatment arm, visit, country, and treatment-by-visit interaction term. Additionally, covariates such as the CAP score at baseline and the interaction between baseline CAP score and visit will be considered in the MMRM. Additional analysis will be done such as a linear mixed model (LMM) with the same fixed categorical effects and covariates as the primary MMRM, and will be used for sensitivity analysis based on multiple imputed datasets (e.g., 100 datasets). The final statistical inference, including parameter estimates and standard error estimates, will follow Rubin’s formula [35]. The statistical test will be two-sided and conducted at a significance level of 5%, resulting in 95% confidence intervals (CIs) (CIs; two-sided). A *p*-value less than 0.05 will be considered statistically significant. The analysis will be based on the modified intention-to-treat (mITT) population.

#### Sample size determination

According to several publications [36–40], the study aimed to determine the targeted difference of interest in CAP scores between the EPL arm and the placebo arm, which was set at 20 dB/m after 6 months. This difference was used in the sample size calculation, assuming a standard deviation of 45 dB/m.

A sample size of 162 patients (*n* = 81 per arm) is required for a 5% two-sided *T*-test to reach 80% power. The primary efficacy population is the mITT population. Assuming a drop-out rate of 15% (such as patients who discontinue the study drug after randomization or certain patients who never started the treatment), 190 patients (intention-to-treat [ITT] population) will be randomized at the beginning of the trial in order to reach the targeted number of 162 patients in the mITT population.

#### Analysis sets

The following analysis sets will be used in the statistical analyses.

• Screened set: All patients who sign the informed consent form.

• Randomization set (ITT): All patients from the screened set who are eligible for the trial based on the defined inclusion and exclusion criteria and are randomly assigned to the EPL arm or the placebo arm.

• mITT: All patients from the randomization set with evaluable CAP scores at baseline and at least one post-baseline CAP measurement and who actually received the randomized treatment (at least 80% of the study drug planned to be given within 6 months). All analyses using the mITT will be done according to the randomized treatment.

• Safety set: Patients who received at least one dose of the randomized treatment. All analyses using the safety set will be done according to the treatment received.

#### Subgroup analyses

Subgroup analyses will be performed depending on the final sample size and data availability for the primary and secondary endpoints. At least 1 subgroup analysis based on CAP score at baseline (< 288 dB/m, ≥ 288 dB/m) will be performed.

#### Adverse events

The incidence of adverse events will be presented by treatment arm in the order of system organ class, with information on high-level group term (HLGT), high-level term (HLT), and preferred term (PT) sorted in alphabetical order. For each treatment arm, the number (*n*) and percentage (%) of patients who experience each adverse event would be recorded. If the same adverse event occurs multiple times in the same patient during a treatment phase, it will only be counted once. The denominator used for calculating the percentages will be the safety population within each treatment arm.

#### Missing data

To impute missing values for post-baseline visits, the approach of utilizing a pattern-mixture model with control-based pattern imputation [41–43] will be adopted.

#### Demographics and baseline characteristics

Descriptive statistics will be used to aggregate data in tables, listings, and figures, as appropriate. Continuous variables will be described with summary statistics, such as *n*, mean, standard deviation (SD), median, the first and third quartiles (Q1 and Q3), and minimum and maximum values.

For each categorical variable, the frequency and percentage in each category will be reported. Percentages will be calculated using the specified denominator in the table based on a given analysis set. Missing data may be included as a separate category in some scenarios, depending on the nature of the variable. Confidence intervals of 95% will be calculated when relevant.

For binary analysis mainly the exploratory endpoints would be considered and for each categorical variable of an exploratory endpoint, a logistic regression or chi-square test will be considered. No formal testing will be done on the demographics.

The statistical analysis will be performed using SAS statistical software, Version 9.4 or higher or SAS Enterprise Guide 7.1 or higher.

### Dissemination plans

The results of this trial may be published or presented at scientific meetings.

## Discussion

The treatment of MASLD generally involves making lifestyle changes, such as modifying diet and engaging in regular exercise to achieve weight loss [44, 45]. Although extensive research and numerous clinical studies have been conducted, there is currently no universally accepted “gold standard” for the treatment of MASLD [1, 23]. More recently, the U.S. Food and Drug Administration approved resmetirom for the treatment of adults with noncirrhotic non-alcoholic steatohepatitis with moderate to advanced liver fibrosis, to be used along with diet and exercise [46]. So far, long-term effects about safety and efficacy of resmetirom treatment are unknown. Furthermore, resmetirom treatment in patients with MASLD did not improve estimates of insulin resistance [47], which may be important to decrease the elevated cardiometabolic risk that is often observed in patients with MASLD [48]. In addition, resmetirom was specifically approved only for patients having steatohepatitis.

EPL have shown to improve liver steatosis in patients with MASLD associated with obesity, diabetes mellitus, and dyslipidemia in several randomized controlled clinical trials, including placebo-controlled studies [28–31, 49]. In addition, EPL also evidently improved liver function tests and treatment adherence and satisfaction as shown in several observational studies. Real-world observational research showed that the administration of EPL lowered levels of aspartate aminotransaminase (AST), alanine aminotransaminase (ALT), and GGT in MASLD patients, regardless of the presence or number of associated comorbidities [50]. In a pooled analysis of 3 observational studies in patients with MASLD, significant improvements in transaminases and lipid levels and steatosis were observed following 24 weeks of EPL treatment, which translated to better treatment adherence and satisfaction [51].

However, the available evidence regarding the efficacy of EPL as an adjunctive MASLD treatment would need an objective assessment of fatty liver changes as well as an assessment of subjective improvement as measured by symptoms score and for the first time by validated QoL score and satisfaction and adherence to treatment. Therefore, to explore further aspects of EPL in the treatment of MASLD associated with T2DM and/or hyperlipidemia and/or obesity, this randomized controlled clinical trial has been designed to gain scientific data on the physical, functional, and QoL-related outcomes for patients.

Previous clinical studies utilized invasive techniques such as liver biopsy and noninvasive techniques such as transabdominal ultrasonography and FibroScan for the detection and measurement of steatosis and fibrosis in MASLD patients treated with EPL. However, these noninvasive techniques are effective only if the steatosis is greater than 30% [52]. In this study, we utilize a noninvasive approach by transient elastography (FibroScan) to evaluate the severity of MASLD by measuring both steatosis (CAP evaluation) and fibrosis (LSM evaluation) [19]. The CAP has shown high sensitivity in detecting low-grade steatosis, defined as fat deposition of 10% or higher. Therefore, the CAP provides a standardized, noninvasive measure for assessing the degree of hepatic steatosis [17, 53]. This will help in the assessment and continuous monitoring of the primary outcome in changes from baseline to 6 months to observe whether there is regression of the disease conditions (such as fibrosis, MASH, and steatosis).

For the first time, this trial will also evaluate changes of QoL parameters, as measured by the CLDQ-MASLD/MASH, a validated questionnaire specific for MASLD/MASH.

In this trial, to minimize the placebo effect we have created an optimized timeline of visits by the investigator to ensure appropriate trial intervention. We will also try to adopt 2016 EASL guidelines [32] for standard-of-care parameters such as diet and physical exercise across all trial sites. Patients participating in the clinical trial would be monitored for safety and medical care throughout the trial period.

The study has a limitation of excluding patients who consumed alcohol, > 20 g/day in women and > 30 g/day in men, i.e., patients with MetALD. Future studies might explore the potential of EPL in MetALD patients.

In summary, our study aims to utilize a rigorous design to comprehensively explore the efficacy of EPL in patients with MASLD on hepatic steatosis and patient-reported indices such as QoL and its safety in MASLD associated with T2DM and/or hyperlipidemia and/or obesity by assessing various outcome measures.

### Trial status

The protocol was reported in accordance with the Standard Protocol Items: Recommendations for Interventional Trials guidelines [33]. Protocol version number and date: LPS16141 (Amendment 2, Version 3.0) and July 15, 2022. Recruitment for the trial began in October 2022 and was completed by August 2023. We were not aware of the fact that, in general, the trial protocol should be submitted for publication before completion of recruitment but considered a submission date before the last patient/last visit.

### Supplementary Information

Additional file 1. SPIRIT checklist.

## Data Availability

Data sharing is not applicable to this article as no datasets are available as the article is based on study design of a clinical trial.

## Abbreviations

ALT: Alanine aminotransaminase
AST: Aspartate aminotransaminase
BMI: Body mass index
CAP: Controlled attenuation parameter
CI: Confidence interval
CLDQ-MASLD/MASH: Chronic Liver Disease Questionnaire–metabolic dysfunction-associated steatotic liver disease/ metabolic dysfunction-associated steatohepatitis
EASL: European Association for the Study of the Liver
eCRF: Electronic case report form
eDiary: Electronic diary
EPL: Essential phospholipids
GCP: Good Clinical Practice
GGT: Gamma-glutamyl transferase
GOS: Global Overall Symptom
HbA1c: Glycated hemoglobin
HDL: High-density lipoprotein
HLGT: High-level group term
HLT: High-level term
HOMA-IR: Homeostatic Model Assessment for Insulin Resistance
ICH: International Council for Harmonisation of Technical Requirements for Pharmaceuticals for Human Use
IRT: Interactive response technology
LDL: Low-density lipoprotein
LSM: Liver stiffness measurement
MedDRA: Medical Dictionary for Regulatory Activities
mITT: Modified intention-to-treat
MMRM: Mixed-effects model with repeated measures
MASLD: Metabolic dysfunction-associated steatotic liver disease
MASH: Metabolic dysfunction-associated steatohepatitis
NYHA: New York Heart Association
PC: Phosphatidylcholine
PT: Preferred term
QoL: Quality of life
T1DM: Type 1 diabetes mellitus
T2DM: Type 2 diabetes mellitus
ULN: Upper limit of normal
